# GCG inhibits SARS-CoV-2 replication by disrupting the liquid phase condensation of its nucleocapsid protein

**DOI:** 10.1038/s41467-021-22297-8

**Published:** 2021-04-09

**Authors:** Ming Zhao, Yu Yu, Li-Ming Sun, Jia-Qing Xing, Tingting Li, Yunkai Zhu, Miao Wang, Yin Yu, Wen Xue, Tian Xia, Hong Cai, Qiu-Ying Han, Xiaoyao Yin, Wei-Hua Li, Ai-Ling Li, Jiuwei Cui, Zhenghong Yuan, Rong Zhang, Tao Zhou, Xue-Min Zhang, Tao Li

**Affiliations:** 1 State Key Laboratory of Proteomics, National Center of Biomedical Analysis, 27 Tai-Ping Road, Beijing, 100850 China; 2 Nanhu Laboratory, Jiaxing, Zhejiang Province 314002 China; 3 Cancer Research Institute of Jilin University, The First Hospital of Jilin University, Changchun, Jilin Province 130021 China; 4 School of Basic Medical Sciences, Fudan University, Shanghai, 200032 China

**Keywords:** Protein aggregation, SARS-CoV-2

## Abstract

Lack of detailed knowledge of SARS-CoV-2 infection has been hampering the development of treatments for coronavirus disease 2019 (COVID-19). Here, we report that RNA triggers the liquid–liquid phase separation (LLPS) of the SARS-CoV-2 nucleocapsid protein, N. By analyzing all 29 proteins of SARS-CoV-2, we find that only N is predicted as an LLPS protein. We further confirm the LLPS of N during SARS-CoV-2 infection. Among the 100,849 genome variants of SARS-CoV-2 in the *GISAID* database, we identify that ~37% (36,941) of the genomes contain a specific trio-nucleotide polymorphism (GGG-to-AAC) in the coding sequence of N, which leads to the amino acid substitutions, R203K/G204R. Interestingly, N^R203K/G204R^ exhibits a higher propensity to undergo LLPS and a greater effect on *IFN* inhibition. By screening the chemicals known to interfere with N-RNA binding in other viruses, we find that (-)-gallocatechin gallate (GCG), a polyphenol from green tea, disrupts the LLPS of N and inhibits SARS-CoV-2 replication. Thus, our study reveals that targeting N-RNA condensation with GCG could be a potential treatment for COVID-19.

## Abstract (web summary)

Coronavirus nucleocapsid (N) protein is important for viral genome packaging and virion assembly. Here the authors show that natural chemical (-)-gallocatechin gallate (GCG) disrupts the liquid–liquid phase separation of N and inhibits SARS-CoV-2 replication.

## Introduction

Human coronaviruses have caused two epidemics, severe acute respiratory syndrome (SARS) and Middle East respiratory syndrome (MERS), since the 21st century. A recently identified new member of the coronavirus genera, SARS-CoV-2, is responsible for the outbreak of COVID-19 pandemic, from which the world is suffering now^1,2^. SARS-CoV-2 shares ~80% sequence similarity with SARS-CoV and entries host cells via the same receptor, angiotensin-converting enzyme 2 (ACE2)^3,4^. As a highly infectious virus, SARS-CoV-2 has rapidly spread worldwide and caused a global health crisis^5^. As of December 1st, 2020, over 63 million people have been confirmed infected and more than 1.4 million deaths have been reported (https://covid19.who.int/). The current treatment for COVID-19 is mainly symptomatic care and supportive^6^. To contain the rapid global spreading of SARS-CoV-2, tremendous efforts have been made to look for efficient treatments for COVID-19. Therefore, a detailed understanding of the molecular events and the underlying mechanisms in the life cycle of SARS-CoV-2, including the viral replication and assembly, is urgently needed.

SARS-CoV-2 is an enveloped, positive-sense RNA virus containing a non-segmented single-stranded RNA genome of ~30,000 nucleotides (nt)^1^. The determination of the full-length genome sequence of SARS-CoV-2 allowed the analysis of the encoded proteins^1,7–9^. 29 proteins were predicted, including 4 structural proteins, spike (S), membrane (M), envelope (E) and nucleocapsid (N). N protein is a highly conserved factor among coronaviruses, for example, the amino acid sequence shares ~90% homology between SARS-CoV-2 and SARS-CoV^10,11^. Similar to N protein of SARS-CoV, the N^SARS-CoV-2^ is a 46 kDa protein with two domains, NH_2_-terminal RNA-binding domain (NTD) and COOH-terminal dimerization domain (CTD)^11,12^. Previous studies of coronaviruses suggested that N protein is an RNA-binding factor that plays a critical role in viral genome packaging and virion assembly^13–15^.

Many RNA-binding proteins, especially those with high percentage of intrinsically disordered region (IDR), were found to be involved in liquid–liquid phase separation (LLPS) process^16–19^. Protein LLPS is a physicochemical event and was recently emerged as a critical mechanism in organizing macromolecules, such as proteins and nucleic acids, into membrane-less organelles^16,20^. These membrane-less cellular compartments were dynamically assembled via LLPS, and conferred important capacities for the cells to initiate biological functions or reactions in response to a number of stresses^20–25^. Upon RNA virus infection, LLPS mediates the formation of stress granules (SGs) and P-bodies (PBs), which are critical for antiviral immunity by inhibiting viral mRNA translation and promoting RNA decay^16–18,26,27^. Interestingly, LLPS was also thought to be critical in viral assembly, including respiratory syncytial viral (RSV)^28^, measles virus (MeV)^29^ and vesicular stomatitis virus (VSV)^30^. A key step during the replication of coronavirus is the association of N protein with viral genomic RNA and the subsequent condensation into higher-order RNA-protein complexes, which initiates the assembly of virions^13,31^. In the current study, by revealing the RNA-triggered LLPS of N protein, we have been able to find the natural chemical, GCG, can disrupt the LLPS of N protein and inhibit the replication of SARS-CoV-2. Our findings not only provide molecular details in SARS-CoV-2 infection, but also present GCG as a lead compound for the development of drug to treat COVID-19.

## Results

### RNA triggers the LLPS of N protein

**Fig. 1:**
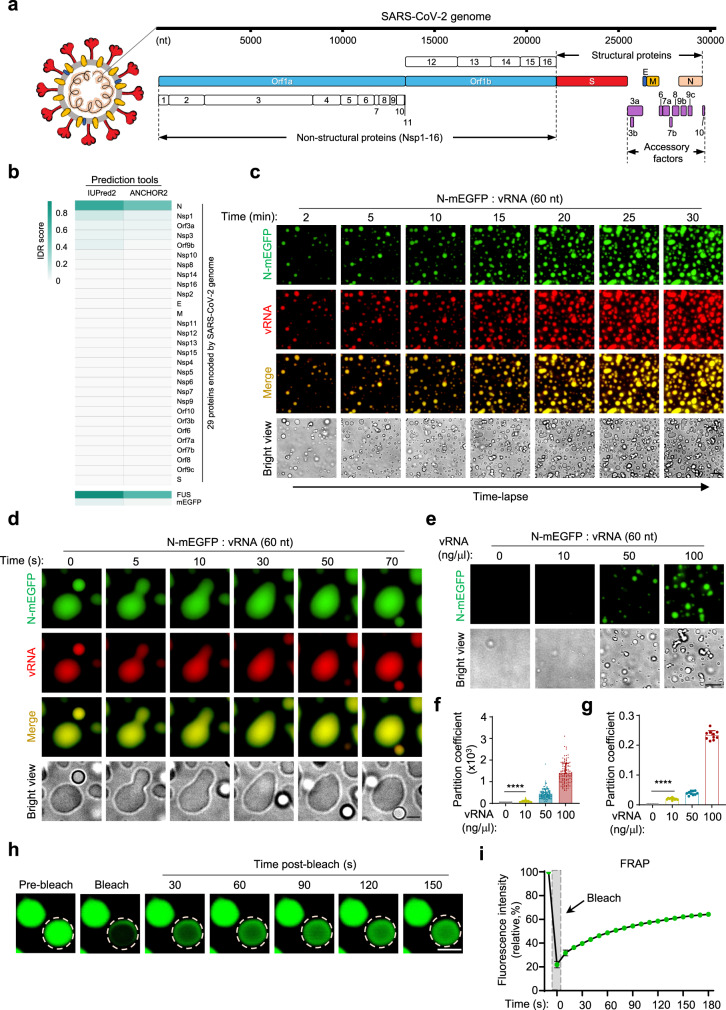
RNA triggers the LLPS of N protein. **a** Schematic drawing of SARS-CoV-2. **b** IDR scores of 29 proteins encoded by SARS-CoV-2 genome. FUS and mEGFP are positive and negative controls, respectively. *IUPred2* and *ANCHOR2* were used as prediction tools. **c** Time-lapse imaging of N-mEGFP protein (20 μM) in the presence of Cy5-labeled 60-nt vRNA (100 ng/μl), scale bar, 10 μm. **d** Representative fluorescent images of N-mEGFP-vRNA (60 nt) condensates fusion from a time-lapse movie, scale bar, 3 μm. **e**–**g** LLPS of N-mEGFP protein (20 μM) in the presence of indicated concentrations of 60-nt vRNA, scale bar, 10 μm (**e**). The partition coefficient of fluorescence intensity per droplet (**f**) and the partition coefficient of total fluorescence intensity in each view (**g**) were calculated. From left to right, *n* = 209, 1170, 1026, 1170 droplets (**f**) from 10 randomly selected views (**g**). **h**, **i** FRAP analysis of vRNA-induced liquid droplets of N-mEGFP protein, scale bar, 2 μm (**h**), and quantification of fluorescence intensity recovery of a photobleached N-mEGFP protein, *n* = 3 biologically independent experiments (**i**). The white dotted circle in **h** indicated the region of photobleaching. 20 μM N-mEGFP protein and 100 ng/μl 60-nt vRNA were used. Error bars, mean with s.d. (**f**, **g**, **i**). Two-tailed unpaired Student’s *t*-test (**f**, **g**), ****P < 0.0001. Data are representative of at least three independent experiments. Source data are provided as a Source Data file.

As protein LLPS has been implicated to play important role in viral assembly^29^, we sought to study the SARS-CoV-2 proteins for their ability to undergo LLPS. Using bioinformatic tools, *IUPred2*, *ANCHOR2, PSPredictor, catGranule, P-Score*, *and PLACC*^32–36^, we analyzed the LLPS ability of each of the 29 proteins encoded by SARS-CoV-2 genome. Only N protein was predicted as an LLPS protein (Fig. 1a, b, Supplementary Fig. 1, 2a and Supplementary Data 1). The known LLPS protein, RNA-binding protein fused in sarcoma (FUS)^19^, and a highly structured, non-LLPS protein, mono-EGFP (mEGFP)^37^, were respectively served as positive and negative controls for the analysis. To further understand the LLPS pattern of N, we analyzed the amino acids and charge distribution using *R* + *Y* and *DDX4-like* predictors^38,39^. We found that N protein exhibited the similar pattern of charged residues as *DDX4-like* proteins (Supplementary Fig. 2b, c).

To further study the LLPS of N protein, we first purified the mEGFP-tagged recombinant N protein and confirmed its RNA-binding capacity with electrophoretic mobility shift assay (EMSA) (Supplementary Fig. 3a, b). When N was incubated with different RNAs, including fragments of SARS-CoV-2 genomic RNAs [a 229-nt 3′ untranslated region (UTR), 229-bp double-stranded RNA (dsRNA) of the 3′ UTR, a 55-nt RNA segment from 5′ UTR or a 60-nt RNA segment from the *Nsp1* coding sequence] and the synthetic analog of dsRNAs, polyinosinic-polycytidylic acid [poly(I:C)] and 5′ppp-dsRNA. We found that RNAs triggered the robust LLPS of N protein both in vitro and in vivo (Supplementary Fig. 3c, d). Using time-lapse microscopy, we observed the dynamic process of RNA-triggered LLPS of N. RNAs formed liquid condensates with N quickly (Fig. 1c and Supplementary Movie 1) and the smaller N-RNA droplets can fuse into bigger ones (Fig. 1d and Supplementary Movie 2), which is a hallmark of protein LLPS^40^. The N-RNA condensation was formed in a concentration-dependent manner (Fig. 1e–g and Supplementary Fig. 4a–c). We further determined the favorable pH (Supplementary Fig. 4d–f), salt concentrations (Supplementary Fig. 4g–i), and RNA lengths for RNA-induced LLPS of N protein (Supplementary Fig. 4j, k). With fluorescence recovery after photobleaching (FRAP) experiments, we showed that the photo-bleached fluorescence signal of N-RNA droplets can be recovered within seconds (Fig. 1h, i and Supplementary Movie 3). This result suggested that the condensates dynamically and rapidly exchange molecules with the environment, which is another feature of protein LLPS^20^. Collectively, these data confirmed that RNA induces the LLPS of N protein.

### N undergoes LLPS in vivo

**Fig. 2:**
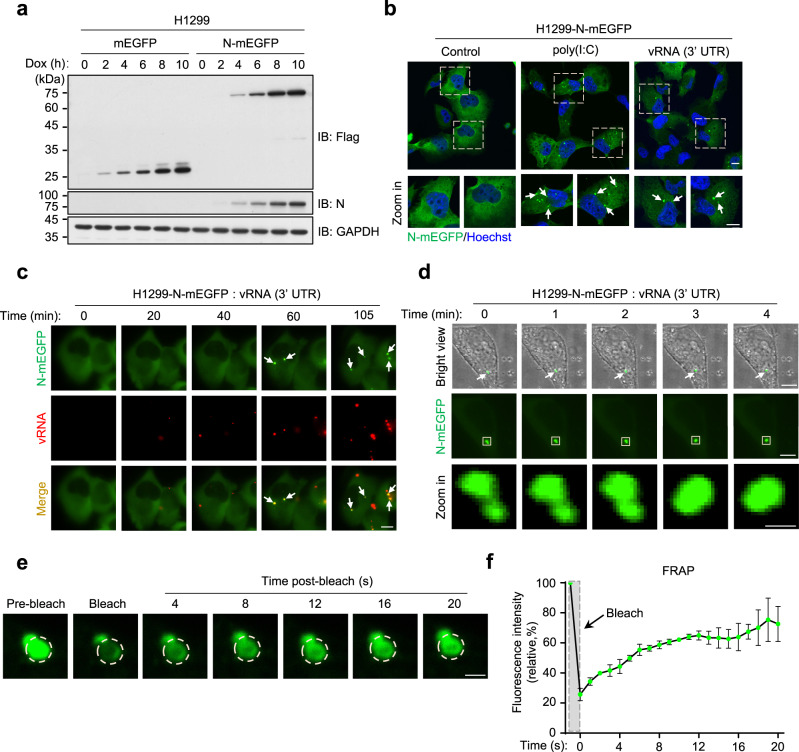
N undergoes liquid–liquid phase separation in vivo. **a** Immunoblot analysis of the Dox-induced expression of Flag-tagged mEGFP and N-mEGFP protein in H1299 cells. **b** Representative fluorescent images of H1299 cells stimulated with 1 μg/ml poly(I:C) or vRNA (3′ UTR). Hoechst (blue), nuclear staining. Scale bar, 10 μm. **c** Time-lapse imaging of N-mEGFP protein *foci* in H1299 cells stimulated with 1 μg/ml Cy5-labeled vRNA (3′ UTR). Scale bar, 10 μm. **d** Liquid droplets fusion of N-mEGFP protein in H1299 cells stimulated with 1 μg/ml poly(I:C). The white square indicated the region of fusion. Scale bars, 5 μm (top, middle), 0.5 μm (bottom). **e** FRAP analysis of liquid droplets of N-mEGFP protein in H1299 cells stimulated with 1 μg/ml poly(I:C). The white dotted circle indicated the region of photobleaching. Scale bar, 1 μm. **f** The quantification of fluorescence intensity recovery of a photobleached N-mEGFP protein in H1299 cells, *n* = 3 biologically independent experiments. Dox (100 ng/ml) was used in H1299 cells in this work unless otherwise indicated. Error bars, mean with s.d. Data are representative of at least three independent experiments. Source data are provided as a Source Data file.

We next investigated the LLPS of N in vivo. We constructed a Doxycycline hyclate (Dox)-inducible N-expressing H1299 cell line (Fig. 2a). Transfection of N-expressing cells with poly(I:C) or the vRNA (3′ UTR), which is shared by all the sub-genome mRNAs^8^, resulted in the formation of N protein condensates (Fig. 2b). Using a Cyanine 5 (Cy5)-labeled vRNA (3′ UTR), we confirmed that the transfected RNA formed condensations with N in cells (Fig. 2c and Supplementary Movie 4). Importantly, the fusion of N-RNA condensates in cells were also observed (Fig. 2d and Supplementary Movie 5). We further performed the FRAP experiment in cells and showed the active molecule-exchanging process of the N-RNA condensates in vivo (Fig. 2e, f and Supplementary Movie 6). These data indicated that the N-RNA condensates in cells were formed via LLPS.

### The LLPS of different N variants

**Fig. 3:**
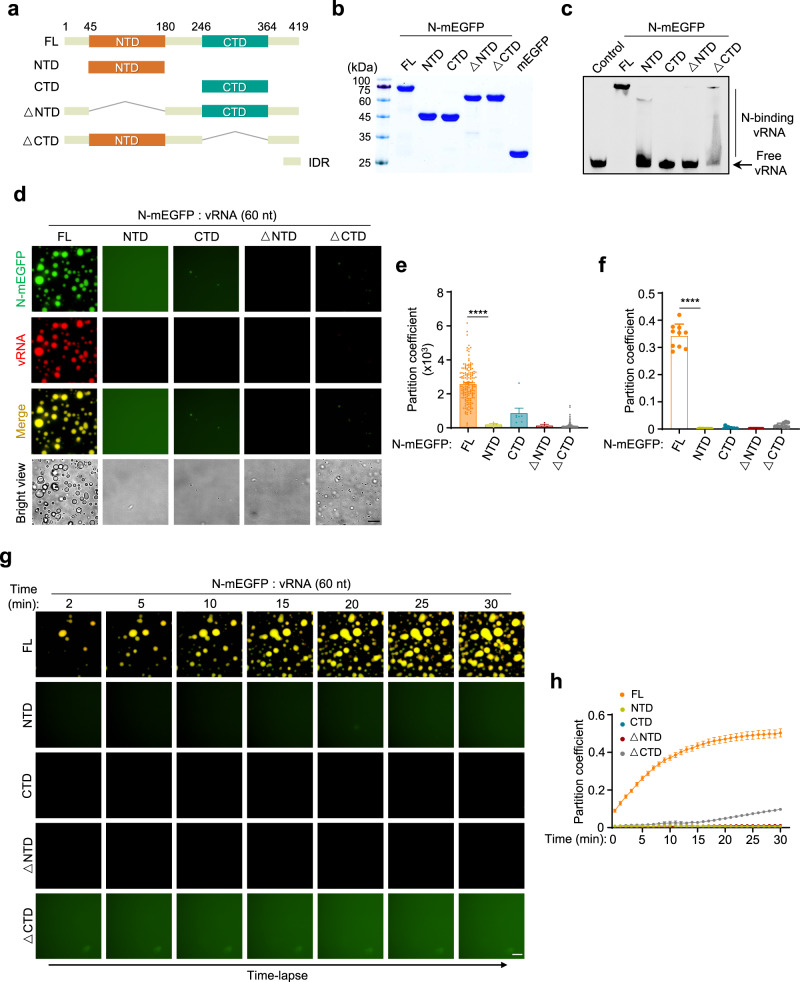
The LLPS of different N variants. **a** Schematic drawing of domains of N protein. **b** Coomassie brilliant blue-stained SDS-PAGE gel of purified mEGFP protein, and full length and truncations of N-mEGFP protein. **c** The RNA-binding capacity of full length and different truncations of N-mEGFP protein (1 μM) was analyzed by EMSA. 55-nt Cy3-labeled vRNA (200 nM) was used as RNA probe. Control, mEGFP protein. **d**–**f** LLPS of full length and different truncations of N-mEGFP protein (20 μM) in the presence of Cy5-labeled 60-nt vRNA (100 ng/μl). The partition coefficient of fluorescence intensity per droplet (**e**) and the partition coefficient of total fluorescence intensity in each view (**f**) were calculated. From left to right, *n* = 1418, 23, 47, 11, 852 droplets (**e**) from 10 randomly selected views (**f**). **g**, **h** Time-lapse imaging of full length and different truncations of N-mEGFP protein (20 μM) in the presence of Cy5-labeled 60-nt vRNA (100 ng/μl) (**g**), and the partition coefficient (*n* = 8 randomly selected views) of total fluorescence intensity (**h**). Scale bars, 10 μm (**d**, **g**). Error bars, mean with s.d. (**e**, **f**) and mean with s.e.m. (**h**). Two-tailed unpaired Student’s *t*-test (**e**, **f**), ****P < 0.0001. Data are representative of at least three independent experiments. Source data are provided as a Source Data file.

By performing the sequence analysis, we found that similar to SARS-CoV, the N protein of SARS-CoV-2 contains two domains, NTD and CTD (Supplementary Fig. 5a, b). The domain definition was also reported recently^11^. To understand whether these structured domains contribute to the LLPS ability, we constructed truncated N variants and purified the recombinant proteins (Fig. 3a, b). Using EMSA, we found that the deletion of any of these domains disrupted the RNA-binding ability of N protein (Fig. 3c). By incubating these variants with the 60-nt viral genomic RNA, we found that none of the truncated N variants can undergo LLPS (Fig. 3d–h and Supplementary Movie 7–11). To further determine the contribution of IDRs in N for LLPS, another variant of only CTD and NTD (connected by a ‘SGGS’ linker) was constructed and prepared (Supplementary Fig. 5c, d). We found that this variant lost the LLPS ability (Supplementary Fig. 5e, f). These data showed that NTD, CTD, and IDRs are all important for the N-RNA binding and the LLPS of N.

### N^R203K/G204R^ gained greater ability to undergo RNA-induced LLPS

**Fig. 4:**
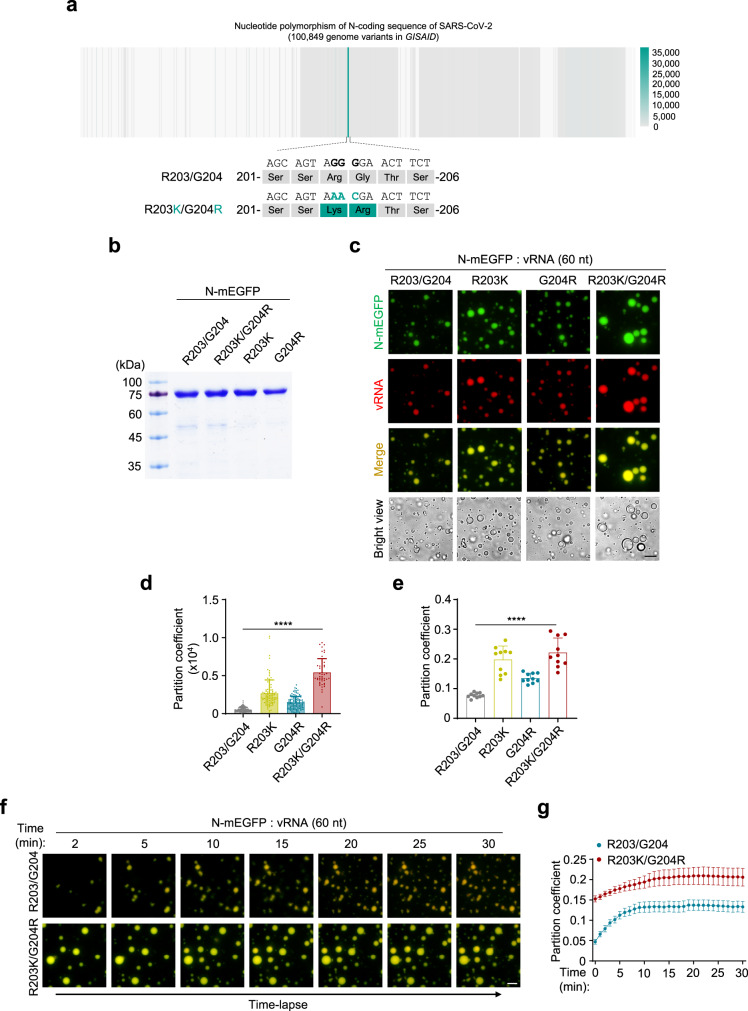
NR203K/G204R gained greater ability to undergo RNA-induced LLPS. **a** Distribution of N gene variants among 100,849 SARS-CoV-2 genomes obtained from *GISAID* database. Colors indicated the nucleotide variability numbers from 100,849 genomes. The high-frequency trio-nucleotide polymorphism variant (GGG-to-AAC) is shown. **b** Coomassie brilliant blue-stained SDS-PAGE gel of purified variants of N-mEGFP protein. **c**–**e** LLPS of different N-mEGFP variants, in the presence of 50 ng/μl 60-nt vRNA (**c**). The partition coefficient of fluorescence intensity per droplet (**d**) and the partition coefficient of total fluorescence intensity in each view (**e**) were calculated. From left to right, *n* = 1232, 803, 897, 431 droplets (**d**) from 10 randomly selected views (**e**). **f**, **g** Time-lapse imaging of N^R203/G204^-mEGFP and N^R203K/G204R^-mEGFP proteins (20 μM) in the presence of Cy5-labeled 60-nt vRNA (40 ng/μl) (**f**), and the partition coefficient (*n* = 8 randomly selected views) of total fluorescence intensity in each view (**g**). Scale bars, 10 μm (**c**, **f**). Error bars, mean with s.d. (**d**, **e**) and mean with s.e.m. (**g**). Two-tailed unpaired Student’s *t*-test (**d**, **e**), ****P < 0.0001. Data are representative of at least three independent experiments. Source data are provided as a Source Data file.

Since the first identification of the genome sequence of SARS-CoV-2^1^, full genomic sequences of this virus from all over the world were continuously submitted to public databases, such as *GISAID* (https://www.gisaid.org). We analyzed 100,849 genome sequences of SARS-CoV-2 from *GISAID* with the attempt to examine the variability of N-coding sequences. Surprisingly, while many nucleotide polymorphisms were found across the full length of the N-coding sequence, a high-frequency trio-nucleotide polymorphism (GGG-to-AAC) was identified in ~37% (36,941) of the genomes (Fig. 4a, Supplementary Fig. 5g and Supplementary Data 2). This GGG-to-AAC variation resulted in the amino acid substitutions, R203K/G204R, in N protein. To examine the effect of this high-frequency variation on the LLPS of N, we prepared the recombinant proteins of these variants, N^R203/G204^, N^R203K^, N^G204R^, and N^R203K/G204R^ (Fig. 4b). When incubated with viral RNA, we found that, interestingly, N^R203K/G204R^ gained greater ability to undergo LLPS (Fig. 4c–g and Supplementary Movie 12, 13). We also analyzed the correlation between the mortality and R203K/G204R polymorphism of N. Our results showed that this polymorphism has little effect on the death ratio reported (Supplementary Fig. 5h). In the future, analysis of patient clinical outcomes and the coupled SARS-CoV-2 genome sequences will provide important evidences regarding the effect of N^R203K/G204R^ polymorphism on the biology of SARS-CoV-2.

### N inhibits RNA-induced *IFN* expression

**Fig. 5:**
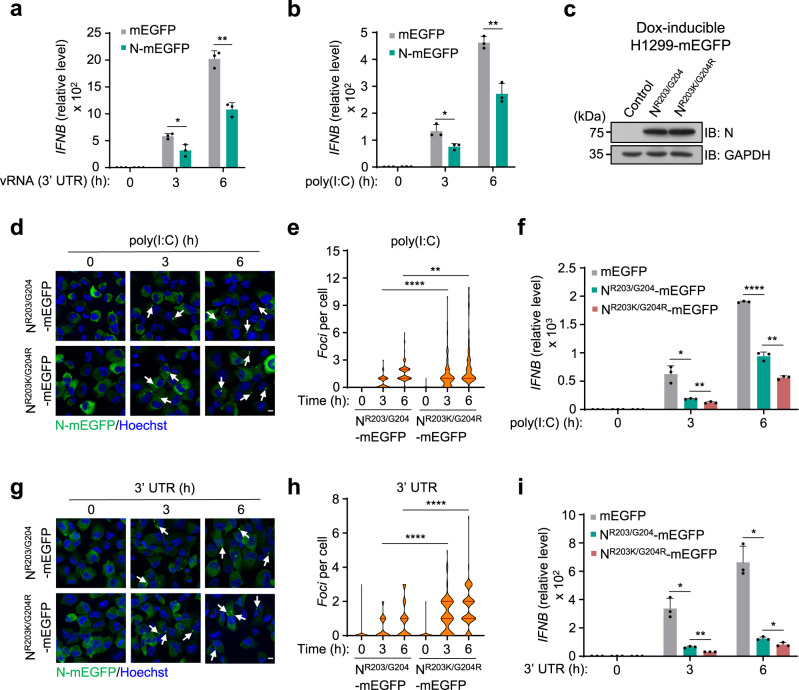
N inhibits RNA-induced IFN expression. **a**, **b** qPCR analysis of *IFNB* expression in H1299 cells stimulated with 500 ng/ml vRNA (3′ UTR) (**a**) or poly(I:C) (**b**), following a 24-h Dox-induced expression of N-mEGFP protein (*n* = 3 biologically independent samples). **c** Immunoblot analysis of the Dox-induced expression of N^R203/G204^-mEGFP and N^R203K/G204R^-mEGFP proteins in H1299 cells. Control, H1299-mEGFP cells. **d**, **e** Representative fluorescent images of H1299 cells stimulated with 1 μg/ml poly(I:C) (**d**). *Foci* of N^R203/G204^-mEGFP and N^R203K/G204R^-mEGFP proteins per cell were quantified, *n* = 100 biologically independent cells (**e**). **f** qPCR analysis of *IFNB* expression in H1299 cells stimulated with 500 ng/ml poly(I:C), *n* = 3 biologically independent samples. **g**, **h** Representative fluorescent images of H1299 cells stimulated with 3 μg/ml vRNA (3′ UTR) (**g**). *Foci* of N^R203/G204^-mEGFP and N^R203K/G204R^-mEGFP proteins per cell were quantified, *n* = 100 biologically independent cells (**h**). **i** qPCR analysis of *IFNB* expression in H1299 cells stimulated with 500 ng/ml vRNA (3′ UTR), *n* = 3 biologically independent samples. Hoechst (blue), nuclear staining (**d**, **g**). Scale bars, 10 μm (**d**, **g**). Violin plots showing *foci* of cells from each group, lines within the plots, with 25th, 50th, and 75th percentiles marked (**e**, **h**). Error bars, mean with s.d. (**a**, **b**, **f**, **i**). Two-tailed unpaired Student’s *t*-test (**a**, **b**, **e**, **f**, **h**, **i**), *P < 0.05, **P < 0.01, ****P < 0.0001. Data are representative of at least three independent experiments. Source data are provided as a Source Data file.

According to a previous study of SARS-CoV, N protein inhibits the virus infection-induced production of interferon (IFN) by interfering with the detection of viral RNA by cellular RNA sensors^41^. To determine the role of SARS-CoV-2 N protein in the RNA-induced expression of *IFN*, we transfected vRNA (3′UTR) or poly(I:C) into the N-expressing and control cells. Our data showed that the expression of N attenuated the intracellular RNA-triggered expression of *IFN* (Fig. 5a, b). We next examined the inhibitory effect of N proteins (both N^R203K/G204R^ and N^R203/G204^) on the RNA-induced expression of *IFN*. We found that the polymorphism of N^R203K/G204R^, which exhibited a higher propensity to undergo LLPS in the presence of RNAs, showed a greater effect on the inhibition of *IFN* expression (Fig. 5c–i). These data indicated that the RNA-triggered phase separation procedure of N protein may shield viral RNAs from host RNA sensors to avoid immune surveillance. Thus, in addition to mediating the package of viral genomic RNA, N may also affect the host antiviral responses. Our data suggested that the inhibitory effect of N is linked with its ability of LLPS.

### GCG inhibits LLPS of N

**Fig. 6:**
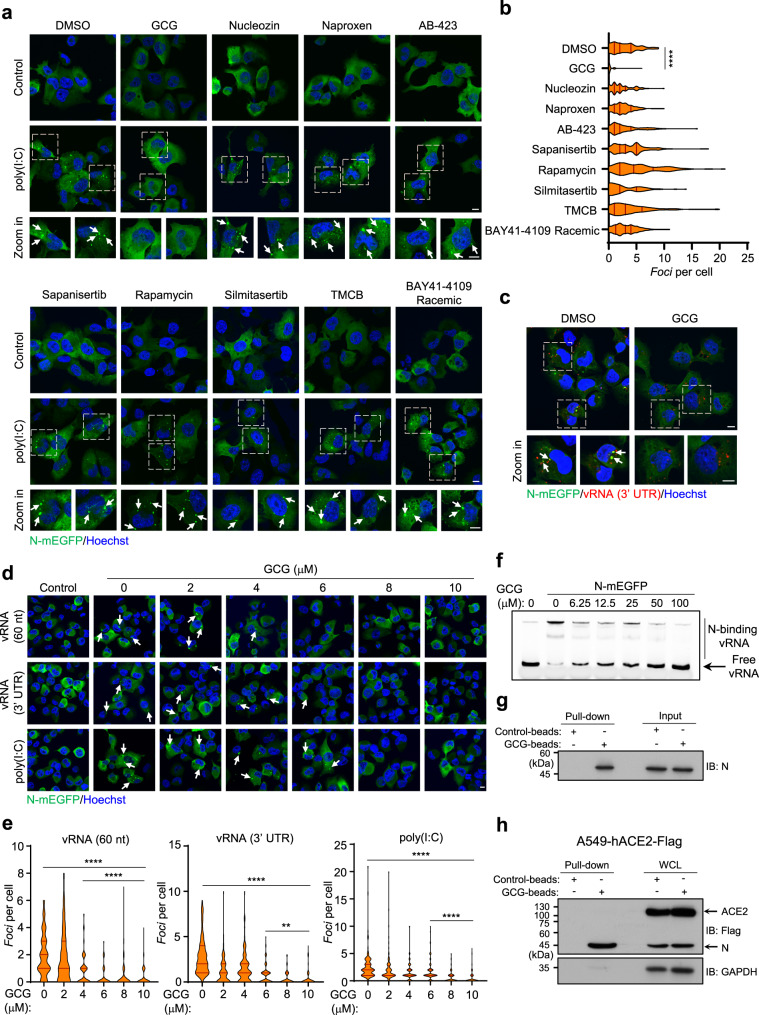
GCG inhibits LLPS of N protein. **a**, **b** H1299 cells with Dox-induced expression of N-mEGFP were transfected with poly(I:C) for 4 h, following a 1-h pretreatment of indicated chemicals. Representative fluorescent images are shown (**a**). *Foci* of N-mEGFP protein per cell were quantified, *n* = 100 biologically independent cells (**b**). 10 μM GCG, 5 μM Nucleozin, 200 μM Naproxen, 100 μM AB-423, 180 μM Sapanisertib, 50 μM Rapamycin, 37 μM Silmitasertib, 100 μM TMCB and 10 μM BAY41-4109 Racemic were used. **c** Representative fluorescent images showed the effect of GCG on LLPS of N-mEGFP protein in H1299 cells stimulated with 1 μg/ml Cy5-labeled vRNA (3′ UTR). **d**, **e** H1299 cells with Dox-induced expression of N-mEGFP were transfected with 1 μg/ml vRNA (60 nt), vRNA (3′ UTR), and poly(I:C), following the pretreatment of indicated concentrations of GCG. Representative fluorescent images were shown (**d**). *Foci* of N-mEGFP protein per cell were quantified, *n* = 100 biologically independent cells (**e**). **f** EMSA showed the effect of GCG on RNA-binding capacity of N-mEGFP protein. Cy3-labeled vRNA (55 nt) was used as RNA probe. **g** Immunoblot analysis of GCG-N interaction in vitro. GCG pull-down assay was performed by GCG-conjugated agarose beads incubated with recombinant N protein. **h** Immunoblot analysis of GCG-N interaction in vivo. GCG pull-down assay was performed by GCG-conjugated agarose beads incubated with lysate of A549-hACE2-Flag cells transfected with pcDNA3.0-Flag-N. Hoechst (blue), nuclear staining (**a**, **c**, **d**). Violin plots showing *foci* of cells from each group, lines within the plots, with 25th, 50th, and 75th percentiles marked (**b**, **e**). Scale bars, 10 μm (**a**, **c**, **d**). Two-tailed unpaired Student’s *t*-test (**b**, **e**), **P < 0.01, ****P < 0.0001. Data are representative of at least three independent experiments. Source data are provided as a Source Data file.

Given that the N-mediated genome organization process is a key step for viral assembly^13,14^, our findings, therefore, provided a potential target for the development of means to combat SARS-CoV-2. With this in mind, we listed several chemicals/drugs that were previously reported to interfere with the N-RNA binding or the self-aggregation of N protein of viruses^42–46^. We also included the chemicals/drugs suggested by a recent report of the proteomics study on SARS-CoV-2^9^ (Supplementary Fig. 6). Next, we transfected poly(I:C) into the N-expressing cells following the pre-treatment of the above chemicals/drugs. GCG blocked the RNA-triggered LLPS of N, while other drugs did not show detectable effect (Fig. 6a). Data from multiple views were calculated and analyzed statistically (Fig. 6b). Using a Cy5-labeled vRNA, we obtained the consistent data (Fig. 6c and Supplementary Fig. 7a). The possibility that GCG affected the transfection efficiency was ruled out (Supplementary Fig. 7b).

To test the cytotoxicity of GCG, different dosages of GCG were used to treat cells, cell viability were measured 48 h after the treatment. Our data showed that the doses of GCG used in our study did not cause an obvious cell death, and the 50% cytotoxicity concentration (CC_50_) was calculated (Supplementary Fig. 7c). We then examined the LLPS of N protein with the application of increasing concentrations of GCG, the results showed that 12.5 μM was sufficient to block the N protein LLPS (Supplementary Fig. 7d, e). We further titrated the concentrations of GCG below 10 μM and found that 6–8 μM were the starting concentrations for GCG to inhibit LLPS of N protein (Fig. 6d, e). By using EMSA, we showed that the presence of GCG significantly impaired the RNA-binding of N protein (Fig. 6f). In addition, by incubating N with GCG, we showed the direct binding of GCG and N protein (Fig. 6g). We further used GCG-beads to pull-down proteins in cells expressing N, and found that GCG selectively bound to N (Fig. 6h). Previously, our group reported that epigallocatechin gallate (EGCG), a structural isomer of GCG inhibited interferon production by disrupting the interaction between GTPase-activating protein-(SH3 domain)-binding protein 1 (G3BP1) and Cyclic GMP-AMP synthase (cGAS)^47^. We then tested the effect of EGCG on blocking the RNA-triggered LLPS of N protein. Interestingly, although these two molecules are isomers, EGCG had much weaker effect on the inhibition of N-RNA condensation (Supplementary Fig. 7f, g). Taken together, GCG directly bound N protein and disrupted N LLPS.

### GCG suppresses SARS-CoV-2 replication

**Fig. 7:**
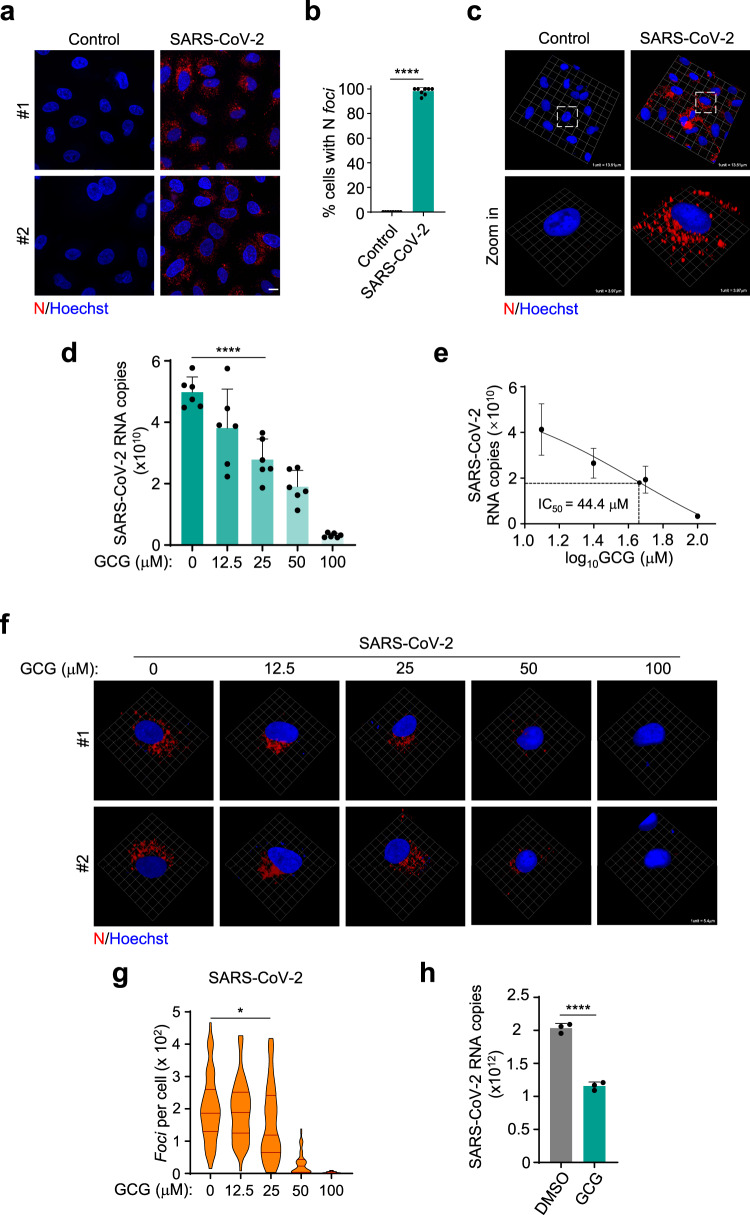
GCG suppresses SARS-CoV-2 replication. **a**, **b** Immunofluorescence analysis of N protein in A549-hACE2-Flag cells infected with SARS-CoV-2 for 24 h (**a**). The percentage of cells with N protein *foci* was quantified, *n* = 8 biologically independent samples, 20 randomly selected views were analyzed in each sample (**b**). Scale bar, 10 μm. **c** 3D images were obtained by Zeiss LSM 880 confocal microscope and reconstituted by *Volocity 6.1.1*. **d**, **e** The inhibitory effect of GCG on the replication of SARS-CoV-2, *n* = 6 biologically independent samples (**d**). IC_50_ was calculated, *n* = 5 biologically independent samples (**e**). The infection was performed after 1-h pretreatment of GCG. **f**, **g** Representative immunofluorescent images showed the inhibitory effect of GCG on SARS-CoV-2 N protein. 3D images were obtained by Zeiss LSM 880 confocal microscope and reconstituted by *Volocity 6.1.1* (**f**). Violin plots showing *foci* of cells (*n* = 50 biologically independent cells) from each group, lines within the plots, with 25th, 50th, and 75th percentiles marked (**g**). **h** Cells were infected with SARS-CoV-2 for 1 h followed by 24-h GCG treatment, *n* = 3 biologically independent samples. Representative images were shown. SARS-CoV-2 was used at an MOI of 1. Hoechst (blue), nuclear staining (**a**, **c**, **f**). Error bars, mean with s.d. (**b**, **d**, **e**, **g**, **h**). Two-tailed unpaired Student’s *t*-test, *P < 0.05, ****P < 0.0001. Data are representative of at least three independent experiments. Source data are provided as a Source Data file.

We next examined whether GCG could inhibit N protein LLPS in the context of SARS-CoV-2 infection. To do so, we obtained the antibody against SARS-CoV-2 N protein, and the specificity of the antibody was verified (Supplementary Fig. 7h, i). We then observed the N LLPS upon SARS-CoV-2 infection, robust formation of N condensates was observed in infected cells (Fig. 7a–c). These data indicated that N protein indeed underwent LLPS during the SARS-CoV-2 infection. By applying GCG treatment on SARS-CoV-2 infected cells, we found that the viral titers were dramatically inhibited (Fig. 7d), and the 50% inhibitory concentration (IC_50_) was calculated (Fig. 7e). The selective index (ratio of CC_50_ to IC_50_) was 3.5. Importantly, the administration of GCG significantly impaired the LLPS of N protein during SARS-CoV-2 infection (Fig. 7f, g). To rule out the possibility that GCG restrict SARS-CoV-2 at the entry step, cells were infected with SARS-CoV-2 for 1 h and then treated with GCG for 24 h. The viral titers were measured, and the results showed that GCG still significantly inhibited the viral replication (Fig. 7h). Together, our data suggested that GCG effectively inhibited SARS-CoV-2 replication and most likely through the disruption of LLPS of N.

## Discussion

SARS-CoV-2 is still raging around the world. The daily confirmed cases are about 491,000 and this number is still increasing. The development of strategies to combat SARS-CoV-2 holds the highest priority. Tremendous efforts have been made to understand the infection of SARS-CoV-2, and the spike-ACE2-mediated viral entry was a major target for many studies^3,4,48,49^. In addition to the viral entry process, it is also critical to understand the details of other molecular events in the life cycle of SARS-CoV-2, such as viral assembly and replication. Recently studies revealed that SARS-CoV-2 carries almost the largest genome in RNA virus family and rapidly replicates in cells^8,50^. The efficient genomic RNA package is therefore important for its replication. Investigation on the mechanisms underlying the assembly of SARS-CoV-2 will be critical in identifying new targets for treating COVID-19. Our work, by unveiling the LLPS of N protein with viral RNA, provided important detailed knowledges of SARS-CoV-2 assembly.

As a physicochemical process, LLPS was more and more realized to be a crucial mechanism that governing the functional organization of macromolecules in numerous biological processes^20,23^. LLPS is believed to be critical in viral assembly^29^. A key step during the replication of coronavirus is the association of N protein with viral genomic RNA and the subsequent condensation into higher-order RNA-protein complexes, which initiates the assembly of virions^13,31^. Our data suggested that in addition to virion assembly, the N-RNAs condensation is also important for shielding viral RNAs from host RNA sensors to avoid host immune surveillance. Interestingly, a recent proteome study identified the protein-protein interaction between N and G3BP1^9^. G3BP1 is a core organizer of SGs assembly^16–18^ and SGs play a crucial role in antiviral responses against RNA viruses^51^. Because G3BP1 mediates the formation of SGs through LLPS^16–18^, N protein may be also involved in SARS-CoV-2 infection-induced formation of SGs through the binding to G3BP1. This involvement could be important for the host to block the translation of SARS-CoV-2 RNAs. On the other hand, N could also hijack G3BP1 or SGs to facilitate virion replication^51,52^.

By analyzing the reported genome sequences, we found that the N^R203K/G204R^ variant, contained by ~37% of the total sequenced SARS-CoV-2 viruses, gained greater ability to undergo RNA-triggered LLPS. Interestingly, N^R203K/G204R^ exhibited a higher propensity to undergo LLPS in the presence of RNAs and showed a greater effect on the inhibition of *IFN* expression. This finding linked the LLPS ability of N protein with its effect on *IFN* inhibition. Although our results showed that N^R203K/G204R^ has little effect on the death ratio of COVID-19 patients, future studies with patient clinical outcomes and the coupled SARS-CoV-2 genome sequences will provide important evidences regarding the effect of N^R203K/G204R^ polymorphism on the biology of SARS-CoV-2. In our study, we have also determined that the acidic microenvironment (pH 6.5) is favorable condition for the RNA-triggered LLPS of N. Although this observation needs to be further investigated, this may propose another perspective for the development of antiviral strategies.

During the revision of this manuscript, a few publications also reported the LLPS of N^53–57^. Our work, however, not only revealed the RNA-triggered LLPS of N as an important molecular event during the life cycle of SARS-CoV-2, but also found that GCG can inhibit SARS-CoV-2 replication by disrupting the LLPS of N. Our findings thus present GCG as a lead compound for the design of anti-SARS-CoV-2 drugs. Given that N protein is a highly conserved protein factor shared by the coronavirus family^58^, targeting N protein represents a novel avenue for drug discovery, not only for SARS-CoV-2, but also for the potential new coronavirus in the future.

## Methods

### Antibodies and reagents

Anti-Flag M2 (F3165, 1:5000) was from Sigma-Aldrich; anti-N (40143-R019, 1:5000) was from Sino-Biological; anti-N (ARG66782, 1:1000) was from Arigo Biolaboratories; anti-β-Actin (20536-1-AP, 1:2000) was from Proteintech Group. Anti-human GAPDH (1:5000) was prepared in our laboratory and generated by immunizing rabbits with human GAPDH protein. Naproxen^42^ (T0855), Nucleozin^43^ (T7330), (-)-Gallocatechin gallate^44^ (T3807), Sapanisertib^9^ (T1838), Rapamycin^9^ (T1537), and Silmitasertib^9^ (T2259) were from TargetMol. AB-423^45^ (HY-112142) was from MedChemExpress. BAY41-4109 Racemic^46^ (S0285) was from Selleck. TMCB^9^ (B7464) was from APExBIO. (-)-Epigallocatechin gallate^47^ (E4143) and Doxycycline hyclate (D9891) were from Sigma-Aldrich. Poly(I:C) (tlrl-pic) and 5′ppp-dsRNA (tlrl-3prna) were from InvivoGen. Full-length SARS-CoV-2 3′ UTR and its complementary RNA were in vitro transcribed and labeled with HyperScribe T7 High Yield Cyanine 5 (Cy5) RNA Labeling Kit (K1062, APExBIO), and the annealed dsRNA (3′ UTR) was from the two transcribed RNAs. Cyanine 3 (Cy3)-labeled 55-nt vRNA (segment of 5′ UTR), Cy5-labeled 10-nt to 60-nt vRNA (segment of *Nsp1*) and 6-carboxy-fluorescein (FAM)-labeled ssDNA were generated by Tsingke Biological Technology. Sequence information is provided in Supplementary Data 3.

### Cell culture and transfection

H1299 (ATCC #CRL-5803) cells were cultured in RPMI-1640 medium containing 10% FBS, 2 mM L-glutamine, 100 U ml^−1^ penicillin, and 100 mg ml^−1^ streptomycin. A549 (ATCC #CCL-185) and A549-hACE2-Flag cells (this paper) were cultured in MCCOY’S 5A containing 10% FBS, 1.5 mM L-glutamine, 100 U ml^−1^ penicillin, and 100 mg ml^−1^ streptomycin. All the cell lines were tested routinely and confirmed to be free of mycoplasma contamination. Transfection of RNAs and ssDNA were performed with Lipofectamine 2000 (Invitrogen). Lenti-virus for the preparation of N-expressing cells were produced in HEK293T (ATCC #CRL-3216) cells.

### Plasmids

cDNA encoding N protein of SARS-CoV-2 was from Sango Biotech. We subcloned the coding sequence of N protein into pcDNA3.0-Flag vector for transient expression, and into pET28a(+) vector linked with C-terminal mEGFP for recombinant protein purification. mEGFP, N-mEGFP, and N^R203K/G204R^-mEGFP were subcloned into pCDX-Tet-On vector with an N-terminal Flag tag and fused with an mEGFP tag at C-terminus for the inducible expression in cells. Five truncations (N^NTD^, N^CTD^, N^▵NTD^, N^▵CTD^, and N^NTD-CTD^) and three mutations (N^R203K^, N^G204R^, and N^R203K/G204R^) were generated from full-length N-mEGFP and subcloned into pET28a(+) vector.

### Cell viability assay

A549-hACE2-Flag cells were seeded into 96-well plates at a density of 10,000 cells per well and incubated with GCG at the indicated concentrations for 48 h. The cell viability was analyzed with CellTiter One Solution Cell Proliferation Assay (MTS) (G3580, Promega) according to the manufacturer’s instruction. 50% cytotoxicity concentration (CC_50_) was calculated by non-linear regression analysis.

### N gene variant identification

Complete SARS-CoV-2 genome sequences (100,849) updated on September 18th, 2020 were downloaded from *GISAID* database (https://www.gisaid.org). To extract all N gene sequences, “Exonerate 2.2.0” software^59^ was used to align N protein-coding sequences to the SARS-CoV-2 genome sequences (*–model protein2 genome: bestfit –score 5 -g y*). The gene sequences of N protein were aligned with MUSCLE 3.8.31^60^ and the annotations and visualizations of mutation sites were processed within R 3.6.0 (https://cran.r-project.org).

### The correlation analysis between mutation frequencies and death ratio

The frequencies of R203K/G204R polymorphism of N protein were calculated with each country and the death ratio information of indicated countries were obtained from WHO website (https://covid19.who.int/). The correlation between the mortality and R203K/G204R polymorphism of N protein was calculated with a linear regression model within R 3.6.0. The subgroup analysis was performed stratified by different continents.

### Sequence alignment analysis

The sequence alignment of SARS-CoV-2 N protein and SARS-CoV N protein (GenBank: AY278741.1) was analyzed and visualized through the msa package within R 3.6.0^61^.

### Phase separation prediction analysis of SARS-CoV-2 proteins

IDR scores of all SARS-CoV-2 proteins were calculated with an *IUPred2A* python script 3.7.3^32^ for each amino acid. A score greater than 0.5 was regarded as intrinsically disordered and the percentage of amino acids with scores greater than 0.5 for each protein was calculated. Modular domains were predicted with InterProScan 5.31-70.0^62^ and we used the predicted results of pfam and SMART for further analysis. Prion-like domains were identified with *PLACC*^36^, *foci*-formation propensity was calculated with *catGranule*^34^, Pi–Pi interactions were analyzed with *P-Score*^35^, and LLPS ability was predicted with an extra machine learning prediction tool *PSPredictor*^33^. The charges of N protein were analyzed according to *DDX4-like* predictor^39^ and the amino acid frequencies of N protein were analyzed according to *R* + *Y* predictor^38^ within R 3.6.0.

### GCG pull-down assay

Pull-down assays were previously described^47^. Briefly, GCG was conjugated with cyanogen bromide (CNBr)-activated agarose beads (C500099, Sangon Biotech). The recombinant N protein (40588-V08B) was from Sino-Biological. A549-hACE2-Flag cells were transfected with pcDNA3.0-Flag-N for 24 h and then lysed with lysis buffer (20 mM Tris-HCl, pH 7.5; 0.5% Nonidet P-40; 250 mM NaCl; 3 mM EDTA and 3 mM EGTA) containing complete protease inhibitor cocktail (04693132001, Roche), followed by centrifugation at 20,000 × *g* for 20 min at 4 °C. The recombinant N protein and the supernatants from cell lysates were incubated with GCG conjugated beads at 4 °C for 6 h. The beads were then washed five times with lysis buffer. The proteins pulled down were examined by 10% SDS-PAGE followed by immunoblotting with indicated antibodies.

### Electrophoretic mobility shift assay (EMSA)

The EMSA was performed to determine RNA-binding capacity of N protein. Recombinant full-length and truncated N-mEGFP proteins were incubated with 55-nt Cy3-labeled vRNA. The mixtures were then applied to an 8% Native-PAGE and the electrophoresis was performed in 0.5 × TBE (Tris-Borate-EDTA) buffer for 1 h at 200 V. The gels were analyzed by ChemiScope 6100 Touch Chemiluminescence imaging system (CLiNX) and ChemiDoc MP Imaging System (Bio-Rad).

### RNA isolation and quantitative PCR (qPCR)

Cells were collected and total RNAs were isolated using TRI reagent (93289, Sigma-Aldrich). Total RNAs (500 ng) were reversed-transcribed to cDNA using PrimeScript RT Master Mix (RR036A, TaKaRa). qPCR was performed with PowerUp SYBR Green Master Mix (A25778, Applied Biosystems), using StepOnePlus Real-Time PCR System (Applied Biosystems) according to the manufacturer’s instructions. Data were analyzed with *StepOnePlus v2.2* software. Primers used are as follows: h*IFNB*-Fwd: 5′- AGGACAGGATGAACTTTGAC-3′; h*IFNB*-Rev: 5′-TGATAGACATTAGCCAGGAG-3′; h*GAPDH*-Fwd: 5′- GAGTCAACGGATTTGGTCGT-3′ and h*GAPDH*-Rev: 5′-TTGATTTTGGAGGGATCTCG-3′. *GAPDH* was used for normalization.

### In vitro phase separation assay

Recombinant N-mEGFP proteins were diluted in phase separation buffer (10 mM Na_3_PO_4_, 150 mM NaCl, pH 6.5), and RNAs were added and mixed in glass-bottom cell culture dishes (801002, NEST) for microscopic observation and image acquirement.

### Fluorescence recovery after photobleaching (FRAP)

Recombinant mEGFP-tagged N proteins were used to performed FRAP assays in vitro. Selected regions were bleached with a 488-nm laser pulse. The fluorescence intensity was collected every 1 s and normalized to the intensity before bleaching. For in vivo FRAP assays, H1299 cells were seeded on the glass bottom cell culture dishes and treated with 100 ng ml^−1^ Dox for the inducible expression of N-mEGFP. After 12-h Dox treatment, the cells were transfected with 1 μg ml^−1^ poly(I:C) for another 6 h. FRAP assays were performed with 488-nm laser pulse and the fluorescence intensity was collected every 0.5 s in vivo and normalized to the intensity before bleaching.

### Protein expression and purification

Constructs for recombinant protein purification were transformed into *E. coli* BL21 (DE3) strain (S106-02, GenStar), and 0.6 mM isopropyl-β-D-1-thiogalactopyranoside (IPTG) (VA20321, GenStar) was used to induce the expression of recombinant proteins. Cells were collected and resuspended in lysis buffer (20 mM Na_3_PO_4_, 1.5 M NaCl, 20 mM imidazole, pH 7.5). Following the sonication and centrifugation, the cleared supernatants were purified with Nickle-coupled agarose beads (G106-01, GenStar) according to the manufacturer’s instructions.

### Formation of N condensates in vivo

H1299 cells were seeded in 24-well plates and treated with 100 ng ml^−1^ Dox for 12 h to induce the expression of N-mEGFP. Then the cells were treated with different chemicals as indicated concentrations, followed by transfection with different RNAs. Cells were fixed with 4% paraformaldehyde for 10–15 min at room temperature, and the nuclei were stained with Hoechst for 10 min. Images were acquired using Zeiss LSM 880 confocal microscope or DeltaVision Deconvolution microscope.

### Virus RNA detection

A549-hACE2-Flag cells were pre-treated with GCG for 1 h, and then infected with SARS-CoV-2 nCoV-SH01 at an MOI of 1 for 24 h, or cells were infected with SARS-CoV-2 for 1 h followed by 24-h GCG treatment. Total RNAs were extracted from cells and viral RNAs were determined using the TaqPath 1-Step RT-qPCR Master Mix (A15299, Thermo Fisher Scientific). Primers and probes used are as follows: SARS-CoV-2-N-Fwd: 5′-GACCCCAAAATCAGCGAAAT-3′; SARS-CoV-2-N-Rev: 5′-TCTGGTTACTGCCAGTTGAATCTG-3′ and SARS-CoV-2-N-Probe: 5′-FAM-ACCCCGCATTACGTTTGGTGGACC-BHQ1-3′. 50% inhibitory concentration (IC_50_) was calculated by non-linear regression analysis.

### Statistical analysis

To determine the partition coefficient of indicated groups, 8 or 10 microscopy images were randomly selected, and the fluorescence intensity was acquired with *Volocity 6.1.1*^63^. Partition coefficient of total fluorescence intensity was calculated as the total fluorescence intensity of droplets divided by the bulk fluorescence intensity of background. Partition coefficient of fluorescence intensity per droplets was calculated as average fluorescence intensity of droplets divide by the bulk fluorescence intensity per pixel of background.

*GraphPad Prism 8.0* was used to perform the statistical analysis. Statistical data are presented as mean with s.d. or s.e.m. as indicated in figure legends. The fluorescence intensity was calculated by *Volocity 6.1.1*. A standard two-tailed unpaired Student’s *t*-test was used for statistical analysis of two groups.

### Reporting summary

Further information on research design is available in the Nature Research Reporting Summary linked to this article.

## Supplementary information

Supplementary Information

Peer Review File

Supplementary Movie 1

Supplementary Movie 2

Supplementary Movie 3

Supplementary Movie 4

Supplementary Movie 5

Supplementary Movie 6

Supplementary Movie 7

Supplementary Movie 8

Supplementary Movie 9

Supplementary Movie 10

Supplementary Movie 11

Supplementary Movie 12

Supplementary Movie 13

Supplementary Data 1

Supplementary Data 2

Supplementary Data 3

Description of Additional Supplementary Files

Reporting Summary

## Data Availability

Complete SARS-CoV-2 genome sequences (100,849) updated on September 18th, 2020 were downloaded from *GISAID* database (https://www.gisaid.org). The death ratio information updated on September 18th, 2020 was obtained from WHO website (https://covid19.who.int/). The annotations and visualizations of mutation sites, the correlation analysis between mutation frequencies and death ratio, the sequence alignment of SARS-CoV-2 N protein and SARS-CoV N protein, and the amino acid frequencies of N protein were analyzed within R 3.6.0 (https://cran.r-project.org). The full-length genome sequence of SARS-CoV-2 nCoV-SH01 strain (accession no. MT121215) and the sequence of SARS-CoV (accession no. AY278741.1) are downloaded from GenBank. Other data related to this study are available from the corresponding author upon reasonable request. Source data are provided with this paper.

## Source data

Source Data

## Code availability

We provided the code that we programmed and used in this study on GitHub at https://github.com/TintingLi/Nprotein_LLPS_analysis.
