# Supplementary material for: GCG inhibits SARS-CoV-2 replication by disrupting the liquid phase condensation of its nucleocapsid protein
Source: Nat Commun. 2021 Apr 9;12:2114. doi: 10.1038/s41467-021-22297-8 (PMC8035206; doi:10.1038/s41467-021-22297-8)
Supplement: Supplementary file 1 — Supplementary Information [file 41467_2021_22297_MOESM1_ESM.pdf]

Supplementary Information

GCG inhibits SARS-CoV-2 replication by disrupting the liquid phase condensation of its nucleocapsid protein

Ming Zhao[1,2,5], Yu Yu[1,3,5], Li-Ming Sun[1,5], Jia-Qing Xing[1], Tingting Li[1], Yunkai Zhu[4], Miao Wang[1], Yin Yu[4], Wen Xue[1], Tian Xia[1], Hong Cai[1], Qiu-Ying Han[1], Xiaoyao Yin[1], Wei-Hua Li[1], Ai-Ling Li[1], Jiuwei Cui[3], Zhenghong Yuan[4], Rong Zhang[4], Tao Zhou[2,*], Xue-Min Zhang[1,4,*] & Tao Li[1,4,*]

Affiliations:

[1]State Key Laboratory of Proteomics, National Center of Biomedical Analysis, 27 Tai-Ping Road, Beijing 100850, China.

[2]Nanhu Laboratory, Jiaxing, Zhejiang Province 314002, China.

[3]Cancer Research Institute of Jilin University, the First Hospital of Jilin University, Changchun, Jilin Province 130021, China.

[4]School of Basic Medical Sciences, Fudan University, Shanghai 200032, China.

[5]These authors contributed equally: Ming Zhao, Yu Yu, Li-Ming Sun.

*Correspondence: tli@ncba.ac.cn (T.L.), zhangxuemin@cashq.ac.cn (X.-M.Z.), tzhou@nanhulab.ac.cn (T.Z.).

Supplementary Fig. 1

Proteins	Prediction tools					
	IUPred2	*ANCHOR2*	*PSPredictor*	*catGranule*	*P-Score**	*PLACC#*
N	0.6850	0.4726	0.9997	1.64764	2.49	48
Nsp1	0.1333	0.0611	0.0003	1.09118	-0.37	0
Orf3a	0.0545	0.0400	0.0052	-0.747331	0.03	0
Nsp3	0.0468	0.0350	0.0177	0.762752	1.48	0
Orf9b	0.0619	0.0103	0.0004	-1.49943	-	0
Nsp10	0.0216	0	0.0016	-0.620617	-	0
Nsp8	0.0101	0	0.0003	-1.0415	-1.34	0
Nsp14	0.0076	0	0.0071	0.125734	1.00	0
Nsp16	0.0034	0	0.0072	0.156967	-0.53	0
Nsp2	0.0016	0	0.0573	0.537649	0.14	0
E	0	0	0.0007	-2.48036	-	0
M	0	0	0.0013	-0.686449	1.11	0
Nsp11	0	0	0.9971	-3.63936	-	0
Nsp12	0	0	0.0079	0.507347	1.04	0
Nsp13	0	0	0.0391	0.0434687	1.44	0
Nsp15	0	0	0.0013	0.411294	-0.70	0
Nsp4	0	0	0.0006	-0.331953	0.30	0
Nsp5	0	0	0.0002	0.240344	1.33	0
Nsp6	0	0	0.0033	-1.21645	-1.13	0
Nsp7	0	0	0.0004	-2.24765	-	0
Nsp9	0	0	0.0012	-0.399386	-	0
Orf10	0	0	0.2228	-2.62158	-	0
Orf3b	0	0	0.0118	-3.46581	-	0
Orf6	0	0	0.001	-2.22528	-	0
Orf7a	0	0	0.0015	-1.83949	-	0
Orf7b	0	0	0.0122	-4.10927	-	0
Orf8	0	0	0.0009	-1.43373	-	0
Orf9c	0	0	0.0031	-2.99986	-	0
S	0	0	0.071	0.839316	1.39	0
FUS	0.9316	0.5608	0.9999	5.75	13.96	239
mEGFP	0.0711	0.0209	0.0047	1.10049	-0.17	0

Supplementary Fig. 1 | LLPS prediction of SARS-CoV-2 proteins. IDR analysis of 29 proteins encoded by SARS-CoV-2 genome.

* Protein sequence analyzed by P-Score should be at least 140 residues.

# VITmaxrun scores were used in PLACC analysis. VITmaxrun: maximum length of consecutive PrD state in Viterbi parse.

Supplementary Fig. 2

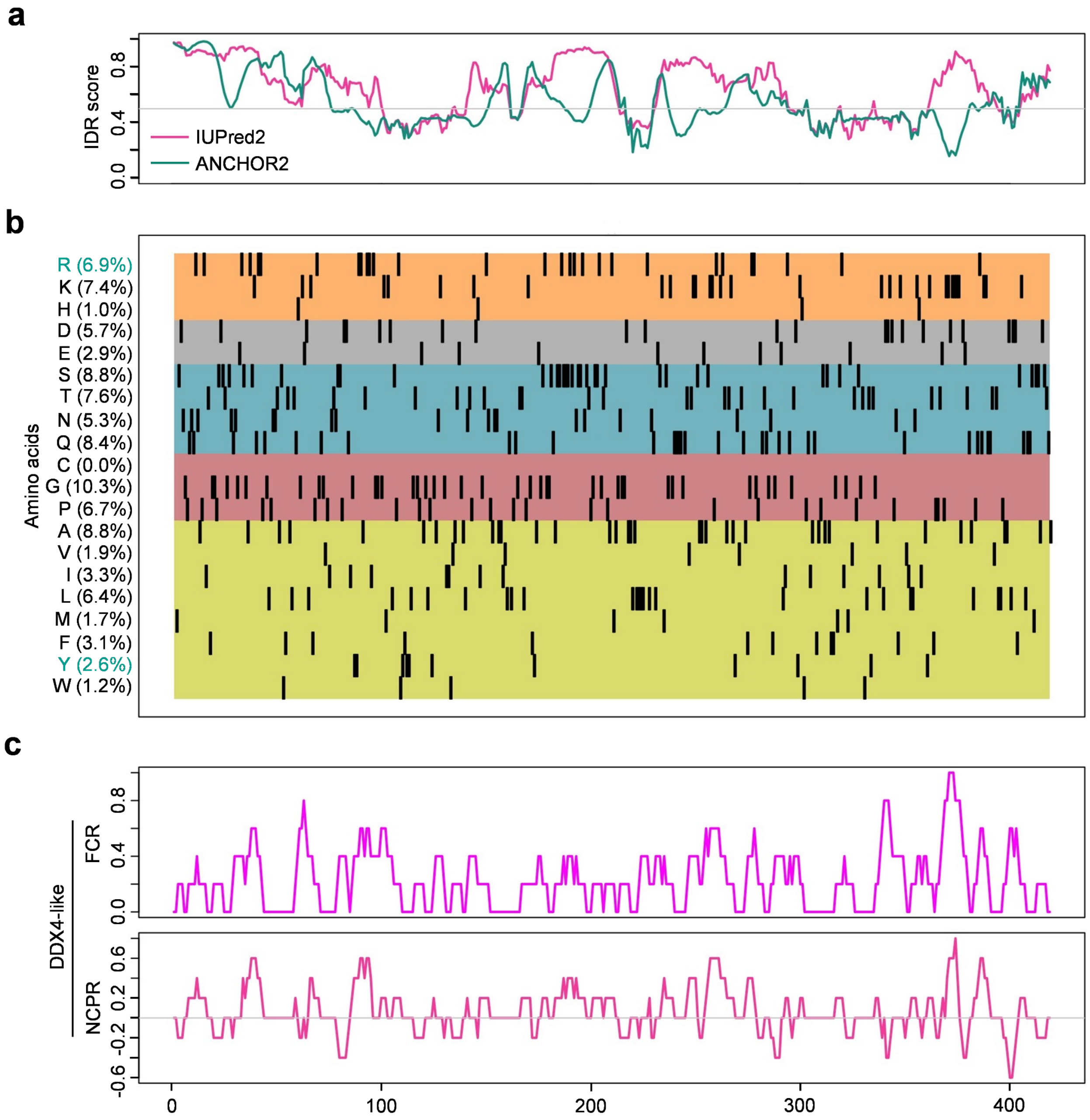

Supplementary Fig. 2 | LLPS pattern analysis of N protein. a, IDR prediction of N protein by *IUPred2* (pink) and *ANCHOR2* (green) methods. **b**, Amino acids frequencies of N protein were analyzed. **c**, Charges of N protein were analyzed. FCR: fraction of charged residues. NCPR: net charge per residue.

Supplementary Fig. 3

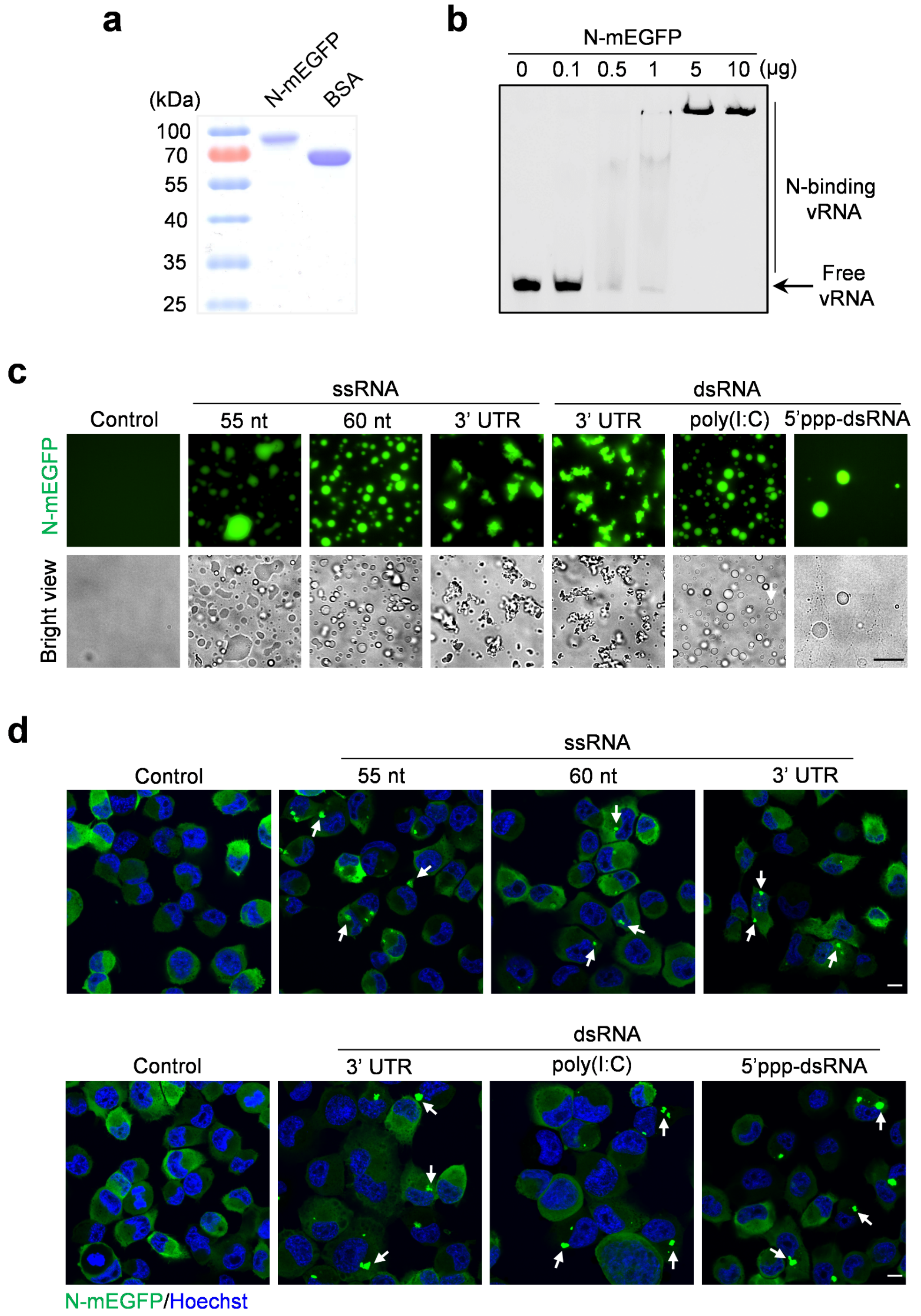

Supplementary Fig. 3 | RNA triggers the LLPS of N protein. a, Coomassie brilliant blue stained SDS-PAGE gel of purified N-mEGFP protein. **b**, The RNA-binding capacity of N-mEGFP protein at indicated amounts was analyzed by EMSA. 200 nM Cy3-labeled vRNA (55 nt) was used as RNA probe. **c**, LLPS of N-mEGFP protein (20 μM) with indicated RNAs (100 ng/μl) treatment. **d**, H1299-N-mEGFP cells were stimulated with indicated RNAs. 3 μg/ml 55-nt vRNA, 3 μg/ml 60-nt vRNA and 1 μg/ml 3' UTR vRNA were used as ssRNA. 3 μg/ml annealing 3' UTR, 1 μg/ml poly(I:C) and 3 μg/ml 5'ppp-dsRNA were used as dsRNA. Hoechst (blue), nuclear staining (**d**). Scale bars, 10 μm (**c**,**d**). Data are representative of at least three independent experiments. Source data are provided as a Source Data file.

Supplementary Fig. 4

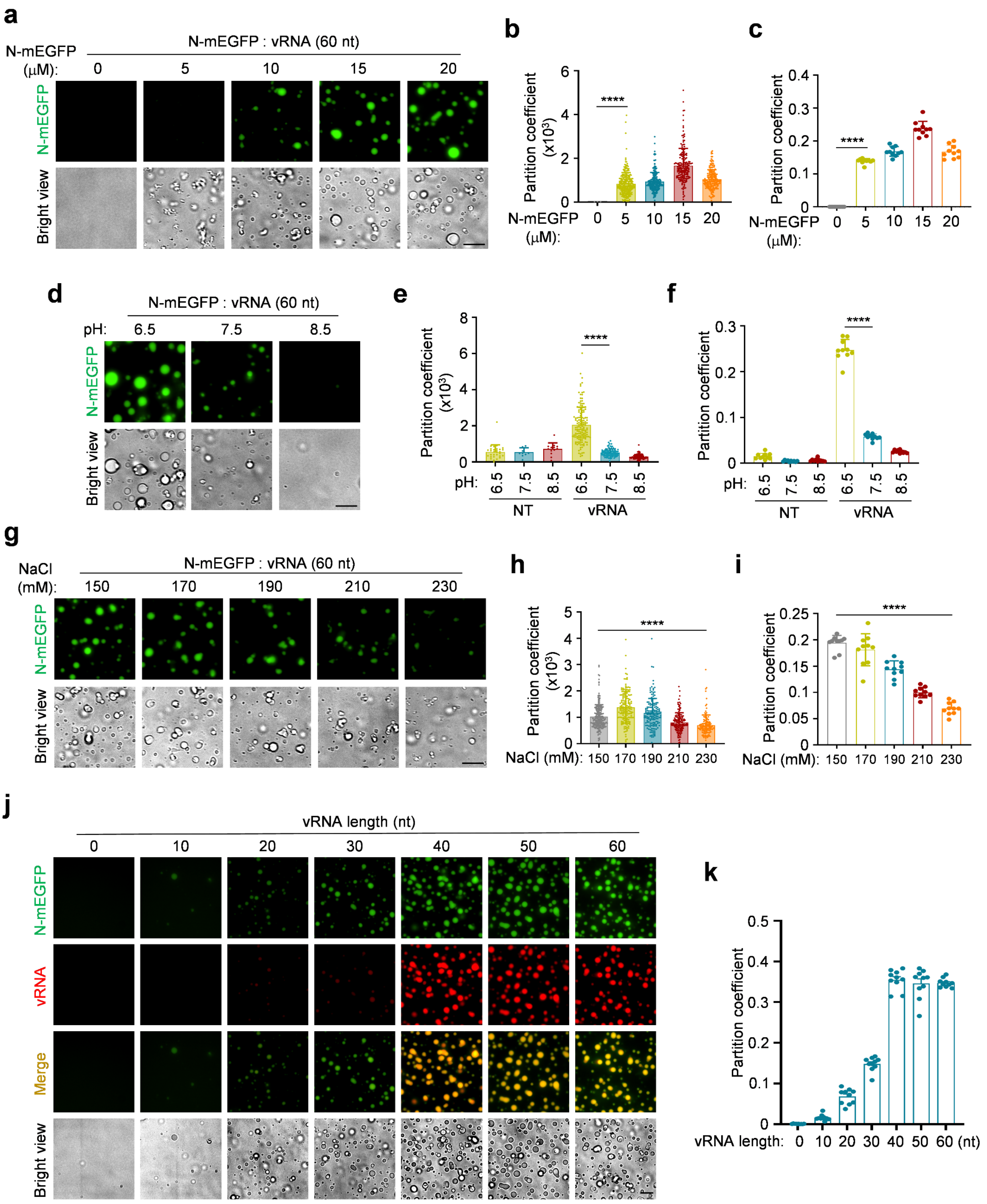

Supplementary Fig. 4 | RNA triggers LLPS of N protein. a-**c**, LLPS of N-mEGFP protein at indicated concentrations in the presence of 60-nt vRNA (100 ng/μl) (**a**). The partition coefficient of fluorescence intensity per droplet (**b**) and the partition coefficient of total fluorescence intensity in each view (**c**) were calculated. From left to right, n = 6, 1991, 2011, 1625, 1673 droplets (**b**) from 10 randomly selected views (**c**). **d**-**f**, LLPS of N-mEGFP protein (20 μM) in the presence of 60-nt vRNA (100 ng/μl), with reaction buffer of indicated pH values (**d**). The partition coefficient of fluorescence intensity per droplet (**e**) and the partition coefficient of total fluorescence intensity in each view (**f**) were calculated. From left to right, n = 217, 69, 83, 1362, 809, 897 droplets (**e**) from 10 randomly selected views (**f**). NT, non-treated. **g**-**i,** LLPS of N-mEGFP protein (20 μM) in the presence of 60-nt vRNA (100 ng/μl), with reaction buffer containing indicated concentrations of NaCl (**g**). The partition coefficient of fluorescence intensity per droplet (**h**) and the partition coefficient of total fluorescence intensity in each view (**i**) were calculated. From left to right, n = 2046, 1385, 1285, 1263, 1053 droplets (**h**) from 10 randomly selected views (**i**). **j**,**k**, LLPS of N-mEGFP protein (20 μM) induced by indicated lengths of vRNAs (5 μM) (**j**). The partition coefficient (n = 10 randomly selected views) of total fluorescence intensity in each view (**k**) were calculated. Scale bars, 10 μm (**a**,**d**,**g**,**j**). Error bars, mean with s.d. (**b**,**c**,**e**,**f**,**h**,**i**,**k**). Two-tailed unpaired Student's *t*-test (**b**,**c**,**e**,**f**,**h**,**i**), ****$P < 0.0001$. Data are representative of at least three independent experiments. Source data are provided as a Source Data file.

Supplementary Fig. 5

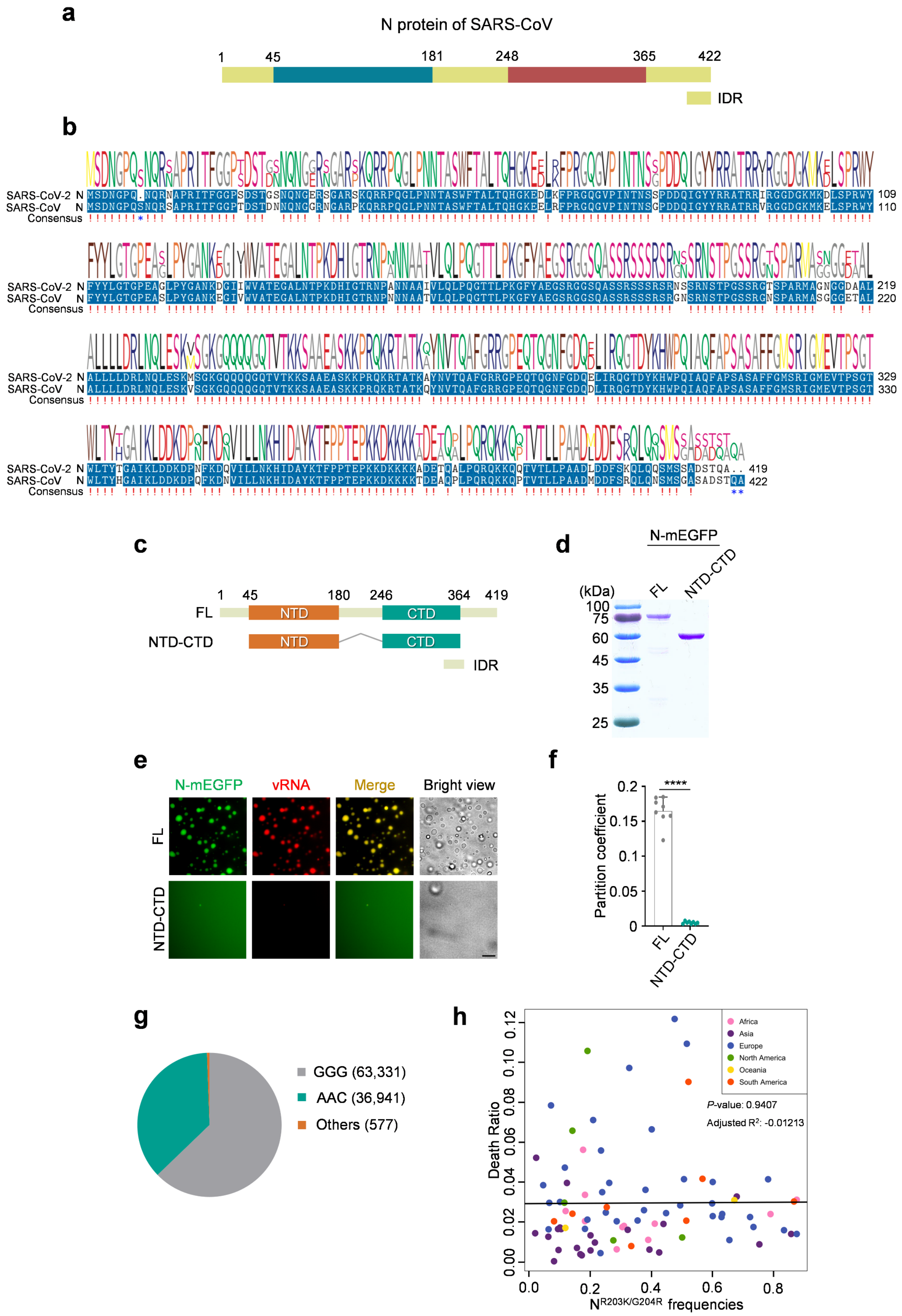

Supplementary Fig. 5 | The LLPS of different N variants. a, Schematic drawing of SARS-CoV N protein. **b**, Sequence alignment of SARS-CoV-2 N protein and SARS-CoV N protein. **c**, Schematic drawing of N^{FL} and $N^{NTD\text{-}CTD}$. **d**, Coomassie brilliant blue stained SDS-PAGE gel of purified full length and NTD-CTD of N-mEGFP protein. **e**,**f**, LLPS of full length and NTD-CTD of N-mEGFP protein (20 μM) in the presence of Cy5-labeled 60-nt vRNA (100 ng/μl). Scale bar, 10 μm (**e**). The partition coefficient (n = 8 randomly selected views) of total fluorescence intensity in each view was calculated. Error bar, mean with s.d.. Two-tailed unpaired Student's *t*-test. $^{****}P < 0.0001$ (**f**). **g**, Pie chart showing the proportion of polymorphism variants. **h**, Correlation analysis between $N^{R203K/G204R}$ frequencies and death ratio. Data are representative of at least three independent experiments. Source data are provided as a Source Data file.

Supplementary Fig. 6

Name	CAS Number	Molecular Formula	Constitutional Formula	Reference
(-)-Gallocatechin gallate	4233-96-9	$C_{22}H_{18}O_{11}$	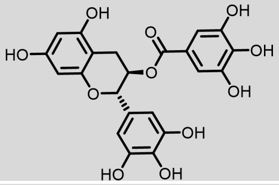	[44]
Nucleozin	341001-38-5	$C_{21}H_{19}ClN_4O_4$	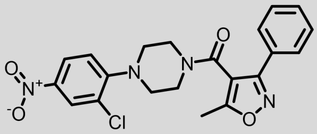	[43]
Naproxen	22204-53-1	$C_{14}H_{14}O_3$	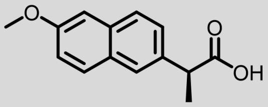	[42]
AB-423	1572510-80-5	$C_{17}H_{17}F_3N_2O_3S$	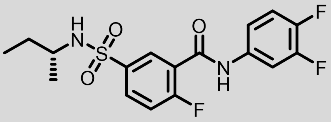	[45]
Sapanisertib	1224844-38-5	$C_{15}H_{15}N_7O$	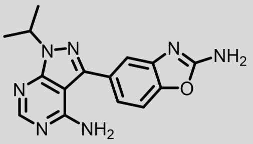	[9]
Rapamycin	53123-88-9	$C_{51}H_{79}NO_{13}$	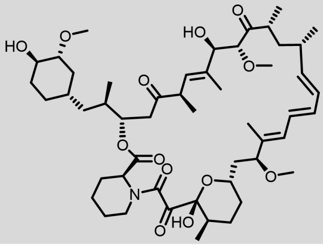	[9]
Silmitasertib	1009820-21-6	$C_{19}H_{12}ClN_3O_2$	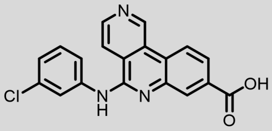	[9]
TMCB	905105-89-7	$C_{11}H_9Br_4N_3O_2$	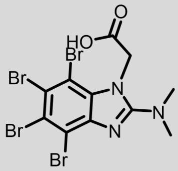	[9]
BAY41-4109 Racemic	298708-79-9	$C_{18}H_{13}ClF_3N_3O_2$	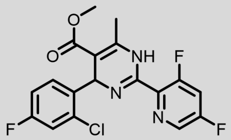	[46]

Supplementary Fig. 6 | GCG inhibits LLPS of N protein. Information of chemicals used in this study.

Supplementary Fig. 7

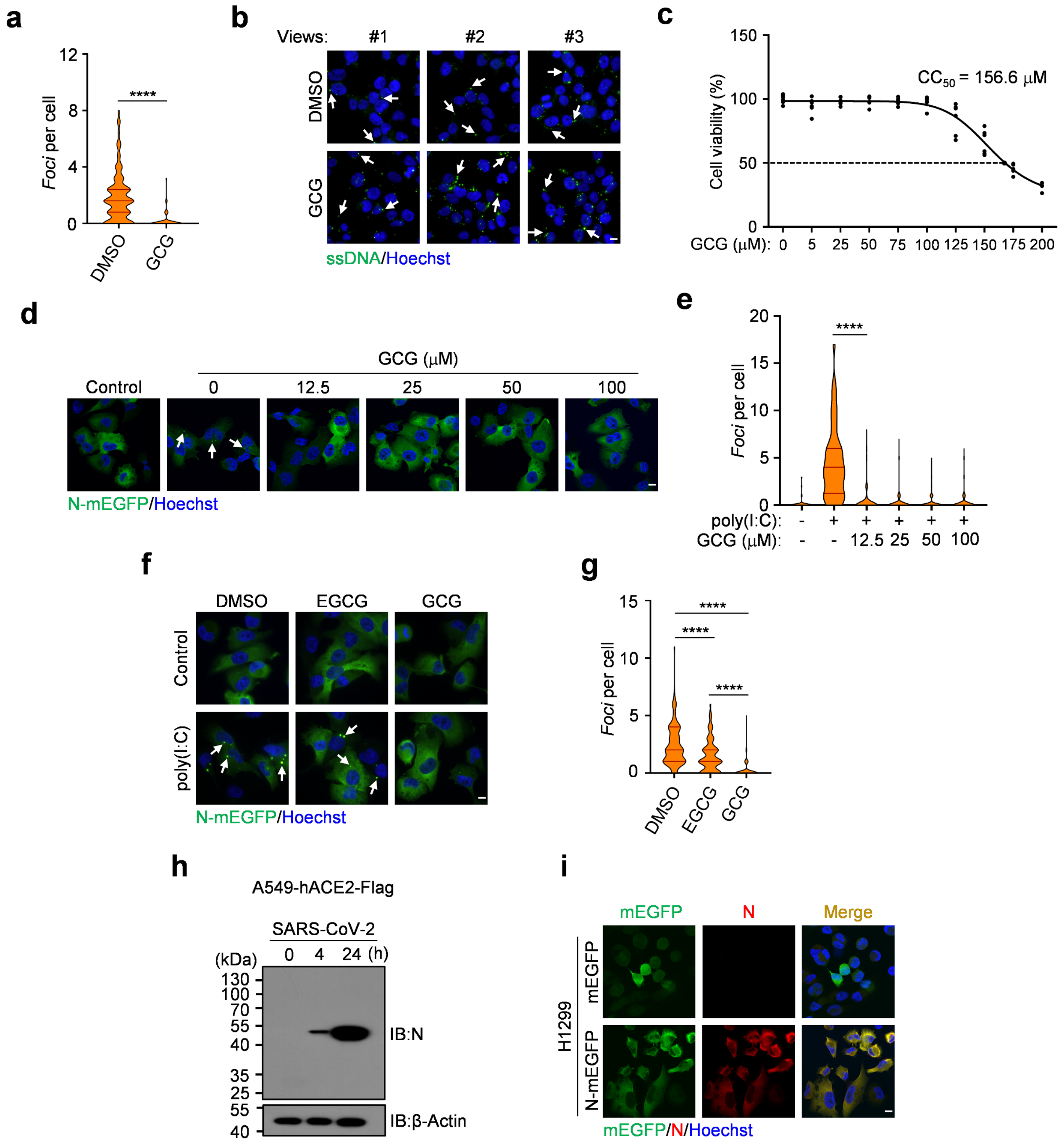

Supplementary Fig. 7 | GCG inhibits LLPS of N and the replication of SARS-CoV-2. a, Quantification of N-mEGFP protein *foci* per cell in **Fig. 6c**, n = 100 biologically independent cells. **b**, The influence of GCG on transfection efficiency. H1299 cells were treated with GCG (10 μM) for 1 h, followed by transfection of 1 μg/ml FAM-labeled ssDNA. Representative three images were shown. **c**. MTS assay on the cell viability of A549-hACE2-Flag cells in the present of indicated concentrations of GCG for 48 h, n = 6 biologically independent samples. CC_{50} was calculated. **d**,**e**, H1299-N-mEGFP cells were transfected with 1 μg/ml poly(I:C), following pretreatment of indicated concentrations of GCG for 1 h. Representative images were shown (**d**). *Foci* of N-mEGFP protein per cell were quantified, n = 100 biologically independent cells (**e**). **f**,**g**, Representative fluorescent images showed the effect of EGCG (10 μM) or GCG (10 μM) on the *foci* formation. H1299-N-mEGFP cells were stimulated with 1 μg/ml poly(I:C), following a 12-hour Dox-induced expression of N-mEGFP protein. Representative images were shown (**f**). *Foci* of N-mEGFP protein per cell were quantified, n = 100 biologically independent cells (**g**). **h**, Immunoblot analysis of N protein expression underlying SARS-CoV-2 infection. **i**, Anti-N antibody was verified by immunofluorescence in H1299-mEGFP and H1299-N-mEGFP cells. Hoechst (blue), nuclear staining (**b**,**d**,**f**,**i**). Scale bars, 10 μm (**b**,**d**,**f**,**i**). Violin plots showing *foci* of cells from each group, lines within the plots, with 25th, 50th and 75th percentiles marked (**a**,**e**,**g**). Two-tailed unpaired Student's *t*-test (**a**,**e**,**g**), $^{****}P < 0.0001$. Data are representative of at least three independent experiments. Source data are provided as a Source Data file.
