# Supplementary material for: GCG inhibits SARS-CoV-2 replication by disrupting the liquid phase condensation of its nucleocapsid protein
Source: Nat Commun. 2021 Apr 9;12:2114. doi: 10.1038/s41467-021-22297-8 (PMC8035206; doi:10.1038/s41467-021-22297-8)
Supplement: Supplementary file 19 — Description of Additional Supplementary Files [file 41467_2021_22297_MOESM19_ESM.docx]

Description of additional supplementary information

Title: Supplementary Movie 1.

Description: Time-lapse imaging of N-mEGFP-vRNA phase separation. N-mEGFP protein (20 μM) and 100 ng/μl Cy5- labeled 60-nt vRNA were used. Corresponds to Fig. 1c.

Title: Supplementary Movie 2.

Description: Fusion of N-mEGFP-vRNA liquid droplets. N-mEGFP protein (20 μM) and 100 ng/μl Cy5-labeled 60-nt vRNA were used. Corresponds to Fig. 1d.

Title: Supplementary Movie 3.

Description: FRAP of N-mEGFP-vRNA liquid droplets. NmEGFP protein (20 μM) and 100 ng/μl Cy5-labeled 60-nt vRNA were used. Corresponds to Fig. 1h.

Title: Supplementary Movie 4.

Description: Time-lapse imaging of N-mEGFP protein foci in H1299 cells. H1299 cells were stimulated with 1 μg/ml Cy5- labeled vRNA (3’ UTR). Corresponds to Fig. 2c.

Title: Supplementary Movie 5.

Description: Fusion of N-mEGFP protein liquid droplets in H1299 cells. H1299 cells were stimulated with 1 μg/ml poly(I:C). Corresponds to Fig. 2d.

Title: Supplementary Movie 6.

Description: FRAP of N-mEGFP-vRNA liquid droplets in H1299 cells. H1299 cells were stimulated with 1 μg/ml poly(I:C). Corresponds to Fig. 2e.

Title: Supplementary Movie 7.

Description: Time-lapse imaging of full length of NmEGFP protein liquid droplets. Recombinant protein (20 μM) and 100 ng/μl Cy5-labeled 60-nt vRNA were used. Corresponds to Fig. 3g.

Title: Supplementary Movie 8.

Description: Time-lapse imaging of NTD of N-mEGFP protein liquid droplets. Recombinant protein (20 μM) and 100 ng/ μl Cy5-labeled 60-nt vRNA were used. Corresponds to Fig. 3g.

Title: Supplementary Movie 9.

Description: Time-lapse imaging of CTD of N-mEGFP protein liquid droplets. Recombinant protein (20 μM) and 100 ng/ μl Cy5-labeled 60-nt vRNA were used. Corresponds to Fig. 3g.

Title: Supplementary Movie 10.

Description: Time-lapse imaging of △NTD of N-mEGFP protein liquid droplets. Recombinant protein (20 μM) and 100 ng/ μl Cy5-labeled 60-nt vRNA were used. Corresponds to Fig. 3g.

Title: Supplementary Movie 11.

Description: Time-lapse imaging of △CTD of N-mEGFP protein liquid droplets. Recombinant protein (20 μM) and 100 ng/ μl Cy5-labeled 60-nt vRNA were used. Corresponds to Fig. 3g.

Title: Supplementary Movie 12.

Description: Time-lapse imaging of R203/G204 of NmEGFP protein liquid droplets. Recombinant protein (20 μM) and 40 ng/ μl 60-nt Cy5-labeled vRNA were used. Corresponds to Fig. 4f.

Title: Supplementary Movie 13.

Description: Time-lapse imaging of R203K/G204R of NmEGFP protein liquid droplets. Recombinant protein (20 μM) and 40 ng/ μl 60-nt Cy5-labeled vRNA were used. Corresponds to Fig. 4f.

Title: Supplementary Date 1.

Description: LLPS prediction of SARS-CoV-2 proteins.

Title: Supplementary Date 2.

Description: Distribution of N gene variants among 100,849 SARS-CoV-2 genomes.

Title: Supplementary Date 3.

Description: RNAs and DNA used in this study.
