# Supplementary material for: GCG inhibits SARS-CoV-2 replication by disrupting the liquid phase condensation of its nucleocapsid protein
Source: Nat Commun. 2021 Apr 9;12:2114. doi: 10.1038/s41467-021-22297-8 (PMC8035206; doi:10.1038/s41467-021-22297-8)
Supplement: Supplementary file 2 — Peer Review File [file 41467_2021_22297_MOESM2_ESM.docx]

REVIEWERS' COMMENTS

Reviewer #1 (Remarks to the Author):

The authors of this manuscript have performed a sizable amount of work to enhance their manuscript and have adequately addressed the concerns of this reviewer. The abstract and grammar throughout the manuscript are much improved. Their additional computational predictions, quantitative analysis of the microscopy images, and cellular experiments strengthen their conclusions. Because of these revisions, this reviewer recommends the manuscript from Zhao, Yu, Sun, et al. be published in Nature Communications.


Jonathon A. Ditlev


Reviewer #2 (Remarks to the Author):

In my view the authors have appropriately responded to the reviewer critiques, and the manuscript has significantly improved. I consider it worthy of publication.


Reviewer #3 (Remarks to the Author):

Following previous reviews, the authors have added substantial new data that sufficiently address previous concerns.
As stated in previously, the subject of this manuscript is of high significance. With the added revisions, this paper is suitable for publication and should be of high interest to a general scientific audience.
